# Comparison of Metabolic Characteristics of Physically Active Individuals with Different Training Habits during Incremental Treadmill Test

**DOI:** 10.3390/ijerph20010070

**Published:** 2022-12-21

**Authors:** Dóra Nagy, Nenad Trunic, Viktória Prémusz, László Krutek, Zoltán Lipcsik, Pongrác Ács

**Affiliations:** 1 Doctoral School of Health Sciences, Faculty of Health Sciences, University of Pécs, 7624 Pécs, Hungary; 2 Institute of Physiotherapy and Sport Science, Faculty of Health Sciences, University of Pécs, 7624 Pécs, Hungary; 3 Physical Activity Research Group, Szentágothai Research Centre, 7624 Pécs, Hungary; 4 Faculty of Physical Culture and Management in Sports, University Singidunum, 11000 Belgrade, Serbia; 5 Medical School, University of Pécs, 7624 Pécs, Hungary

**Keywords:** metabolism, vita maxima test, metabolic flexibility, aerobic training, indirect calorimetry, metabolic syndrome

## Abstract

The number of people engaging in self-conducted regular physical activity is increasing, but the effects of home fitness and individually planned workouts on health and metabolism are unknown. We aimed to examine the effects of regular training conducted without the supervision of professionals on exercise metabolism in our cross-sectional observational study. Forty-five physically active volunteers, classified into three groups, based on the type and frequency of their training (group 1 frequent long-term endurance, group 2 three times per week aerobic training, and group 3 two times per week short aerobic and resistance training), fulfilled a vita maxima incremental treadmill test. Aerobic capacity (VO2max), MET (metabolic equivalent of task), and metabolic responses were examined. The results were evaluated by ANOVA and Bonferroni and Scheffe multiple comparison analysis using Microsoft Excel and SPSS 23 programs. (*p* < 0.05). Significant differences were found between group 1 and 3 in VO2max (*p* = 0.46) and MET (*p* = 0.46) between group 1 and 2, in FatmaxHR (heart rate on maximum fat oxidation) (*p*= 0.04). We concluded self-conducted regular physical activity has positive effects on metabolism and health. Aerobic training performed four times per week showed the most beneficial effects on metabolism and health maintenance. In addition, based on our findings, strength training performed two times per week is recommended.

## 1. Introduction

Regular physical activity (PA) (as defined by the World Health Organization (WHO) as “any bodily movement produced by skeletal muscles requiring energy expenditure”) [1] has a positive impact on the health of physically inactive individuals, even if improving performance is not the primary goal of these activities. Structured exercise training has increasing importance, not only in avoiding chronic illnesses but also in fighting viral infections [2], as we could see in the 2019–2021 COVID pandemic. Putting the focus on physical activity, the WHO updated its recommendation released in 2010, last year. The guidelines highlighted the importance of regular aerobic and muscle-strengthening activities. In the WHO’s recommendation for adults (18–64 years), moderate-intensity aerobic physical activity was increased to up to 300 min, or up to 150 min of vigorous-intensity aerobic physical activity, or an equivalent combination of moderate-intensity and vigorous-intensity activity throughout the week [3]. Sedentary behavior has been linked to negative health outcomes such as all-cause mortality, cancer mortality and the incidence of cardiovascular disease and type 2 diabetes [1].

For measuring and proving the health benefits of regular physical activity and the level of compliance with the recommendations of the WHO, physical activity questionnaires, like the International Physical Activity Questionnaire (IPAQ-HL), are appropriate methods. In their studies, Ács et al. demonstrated IPAQ-HL to be a reasonably valid measure for self-reporting of vigorous, moderate-to-vigorous, and moderate activities [4,5,6,7].

One of the most common risk factors for chronic illnesses and viral infections like COVID-19 syndrome is metabolic syndrome (MS) [8,9]. MS is a precursor of cardiovascular diseases; thus, a high level of aerobic and cardiorespiratory fitness is inversely related to MS development [10,11]. The characteristics of metabolic syndrome are connected to sedentary life, physical inactivity, and nutritional disorders. MS is a severe health condition that results in a higher risk of heart disease, diabetes, stroke, and diseases related to atherosclerosis. Fundamental causes of metabolic syndrome include overweight and obesity, insulin resistance, physical inactivity, and genetic factors [12,13]. In a recent global status report on chronic disease, the WHO stated cardiovascular disease, diabetes, and obesity are responsible for approximately two-thirds of deaths worldwide [3].

Insulin resistance (IR) is associated with fat accumulation and low mitochondrial oxidative capacity in skeletal muscle cells. Phielix et al. examined whether high oxidative capacity could attenuate lipid-induced IR among endurance-trained athletes. Endurance- trained athletes are characterized by high oxidative capacity, higher insulin sensitivity, and elevated intramyocellular lipid level. They found that trained subjects had 32% higher mitochondrial capacity and 22% higher insulin sensitivity (*p* < 0.05 for both). They concluded that long-term exercise training reduced lipid-induced IR [14]. Patients with metabolic syndrome have a decreased capacity to oxidize lipids and an early shift from fat to carbohydrate oxidation during exercise, which is a sign of “metabolic inflexibility” [15,16,17]. The risks caused by MS can be reduced significantly by losing weight, increasing physical activity, and changing diet. Metabolic flexibility (MF) is essential for adaptation to energy resources and requirements. MF can maintain homeostasis during caloric excess or restriction, as well as low and high energy demands, such as exercise. At a molecular level, metabolic flexibility is mainly connected to the mitochondria. Disturbed metabolic flexibility can be linked to several pathological conditions, including type 2 diabetes and cancer [18,19].

Modern nutritional habits are characterized by frequent intakes of calorically concentrated foods, with low nutrient content, leading to disturbed energy homeostasis. Skeletal muscles in healthy people can adapt to fuel preference, which is considered metabolic flexibility [18,19,20]. Muscle fiber type also differs in metabolism and determines the type of energy source, such as the intensity of exercise, gender, training status and training methods, age, diet, and the environmental conditions [21,22,23].

Indirect calorimetry (IC) is a reliable tool that provides minute by minute data of O_2_ consumption and CO_2_ production, both at rest and during physical activity [24]. The energy characteristics and costs of the activity [25] and Maximal Fat Oxidation (MFO), as well as the intensity at which MFO occurs, can be observed during an incremental treadmill test measured by IC [26]. Based on the results of IC, conclusions can be drawn about exercise metabolism and metabolic flexibility [27].

Thanks to the increasing popularity of health promotion and home fitness programs, many people conduct their workouts based on web-based video and educational content, which, according to research by Makai et al. could be an appropriate way to promote a healthy and physically active lifestyle [28]. During the lockdowns of the COVID pandemic, more individuals started to do regular physical activity without any supervision from a trainer or professional, both in Hungary and worldwide [29,30,31]. Since the 3rd wave of the COVID pandemic, 42.51% of the surveyed Hungarians perform physical activities for the purposes of recreation or health maintenance at home, and 33.42% in parks, according to the findings of Ács et al. [32] The increasing number of people engaging in self-conducted regular physical exercise could be a desirable trend; although, the effects of home fitness and individually planned workouts on health and metabolism are unknown.

Therefore, the objective of our study was to examine the effects of regular training conducted without the supervision of professionals on exercise metabolism in a cross-sectional observational study. Cardiovascular and exercise metabolic parameters were measured by indirect calorimetry during an incremental treadmill test performed until exhaustion. We aimed to compare the metabolic adaptation of physical activity with different intensities and frequencies, planned and carried out without professional supervision.

## 2. Materials and Methods

### 2.1. Selection and Description of Participants

This cross-sectional observational study was conducted in Pécs, Hungary, in May 2021, following the third COVID pandemic lockdown. Non-randomized, consecutive sampling was carried out. Forty-five physically active, healthy (defined as having no chronic disease or acute injury that would prevent PA) individuals responded to our call for research participants at the University of Pécs. People whose training duration and intensity met the physical activity criteria of the WHO [1] were considered physically active and were included along with additional criteria, like conducting their training for a minimum of six months consecutively without the supervision of trainers or experts. All the attendees conformed to the inclusion criteria. After the introductory conversation with each of the participants, we classified them into groups based on the type, frequency, and duration of their training sessions. All PA conducted by the participants for health-related reasons were categorized as “training sessions”. The characteristics of their workouts were self-reported. Volunteers also self-reported as being free of any chronic disease, such as MS or diabetes, and acute pain or injury. In Group 1, participants executed training a minimum of four times a week, with low-intensity long-distance running or cycling for 60–90 min (aerobic training) per session. In Group 2, they did the same type of workout three times per week for 35–55 min. Finally, in Group 3, individuals completed 45–60 min of aerobic workouts, (like spinning, spinracing or running) mixed with lightweight or bodyweight resistance training two times per week. Our objective was not to control the entire training process or confirm the reliability of self-reports.

The primary purpose of the study was to determine the metabolic state of the participants and identify differences between the three groups in order to find out which type of physical activity had the most beneficial effects on exercise metabolism. Consequently, we only performed the measurement method once. Consistent with the researchers who examined six months of continuous training, we intended to benefit from the long-term effects of six months of physical activity, which can be observed and studied across all age groups. During this period, participants could also identify and correct deficiencies in training management and dosage (i.e., overtraining and undertraining).

### 2.2. Experimental Procedure

After five minutes of resting 10-lead ECG (Electrocardiography, for identifying test contraindications), participants fulfilled an incremental vita maxima treadmill test till exhaustion, on a Woodway PPS 55 MED treadmill. Cardiorespiratory parameters were recorded with a Cardiosys Plus CAR-02-IA (RHR) cardiovascular analysis system and a Jaeger Masterscreen CPX spirometer. The starting speed of the treadmill test was 8 km/h with zero inclination. We increased speed by 1 km/h and inclination by 3 degrees every three minutes. Aerobic capacity (VO2; mL/kg/min), maximum heart rate (HRmax; Bpm), maximum load (Load; Watt), MET (a calculated value to describe exercise intensity and metabolism), and metabolic characteristics, like respiratory exchange ratio (RER, VCO2/VO2), heart rate at RER1 (1RER; Bpm), maximum fat oxidation (MFO; g/day), heart rate at MFO (FatmaxHR; Bpm) and maximum heart rate percentage at Fatmax (Fatmax%), and the percentage of VO2max at MFO (VO2max%) were examined. Individuals performed the test under the same conditions and at the same time of the day. Participants were asked to continue their everyday nutritional habits and not change their training methods before the test.

The tests were performed following the COVID-19 pandemic lockdown, when more people were training individually without professional supervision. During the restrictions, it was impossible to measure them in person before the training; therefore, we conducted our experimental research with a single test following the lockdowns.

### 2.3. Statistical Analysis

Data were entered in Microsoft Excel and analyzed using IBM SPSS 26.0 program. Descriptive analysis was carried out. The data were expressed as the mean (±SD). Normality of the data was tested using the Kolmogorov–Smirnov test (data was considered normally distributed if *p* < 0.05). A confidence interval of 95% was applied, and *p* value of < 0.05 was considered statistically significant. The results were evaluated by descriptive statistical methods such as one-sample and paired *t*-test, ANOVA, and Bonferroni and Scheffe multiple comparison analysis using Microsoft Excel and SPSS 26 programs. A *p*-value of < 0.05 was considered statistically significant.

## 3. Results

### Demographic Data

No significant differences were found in the participants’ age, body mass, height, and BMI between the groups. Table 1 shows the characteristics of each group.

ANOVA analysis revealed significant differences between the groups for VO2max, MET, FatmaxHr, Fatmax%, and VO2max%, with group 1 producing the highest values for all parameters (0.045; 0.046; 0.004; 0.001, and 0.000, respectively) (*p* < 0.05). No significant differences were detected in HRmax results, in which the highest values were observed in group 3 and the lowest in group 1. The RER1 heart rate value (heart rate at RER (VCO2/VO2) value 1.00) and peak RER data differed between the three groups however, these variations were not statistically significant (*p* = 0.872 and 0.423, respectively) (*p* < 0.05). MFO was higher in group 3 than in group 2 and group 1. The load, calculated in watts, was the highest in group 1, and the smallest in group 3. The differences were not significant (*p* = 0.715 and 0.082) (*p* < 0.05). The results of the incremental vita maxima treadmill test are shown in Table 2. 

Multiple comparison analysis revealed significant differences between group 1 and group 3 in VO2 max values (*p* = 0.46 and 0.41 with Bonferroni and Scheffe tests) and MET parameters (*p* = 0.46 and 0.41 with Bonferroni and Scheffe tests) (*p* < 0.05). We detected a significant difference in FatmaxHR between group 1 and group 2 (*p* = 0.04 and 0.03, respectively) (*p* < 0.05). Comparing the Fatmax% of groups 1 and 2, and groups 1 and 3, significant differences were observed (*p* = 0.03 and 0.02) (*p* < 0.05). Significant differences in VO2% were observed between group 1 and group 2. Table 3 shows significant Bonferroni and Scheffe analysis results.

**Figure 1:**
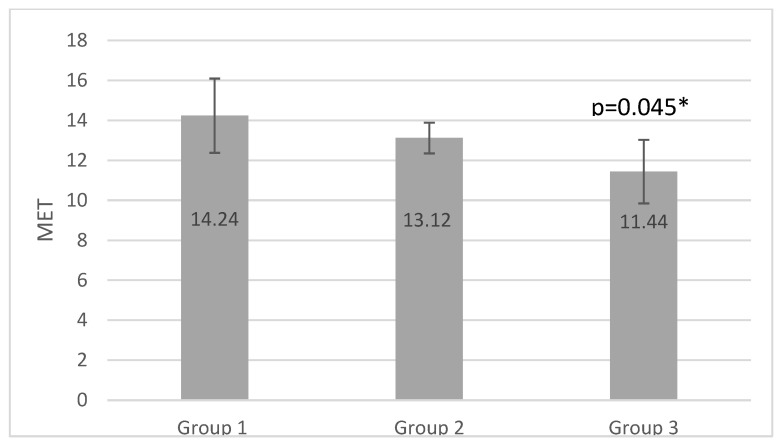
MET results of the groups (* *p* <0.05).

**Figure 2:**
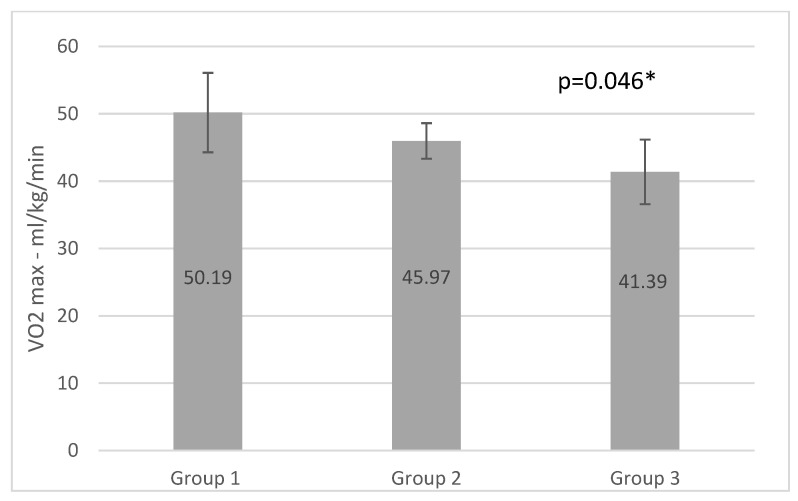
VO2max results of the groups (* *p* <0.05).

**Figure 3:**
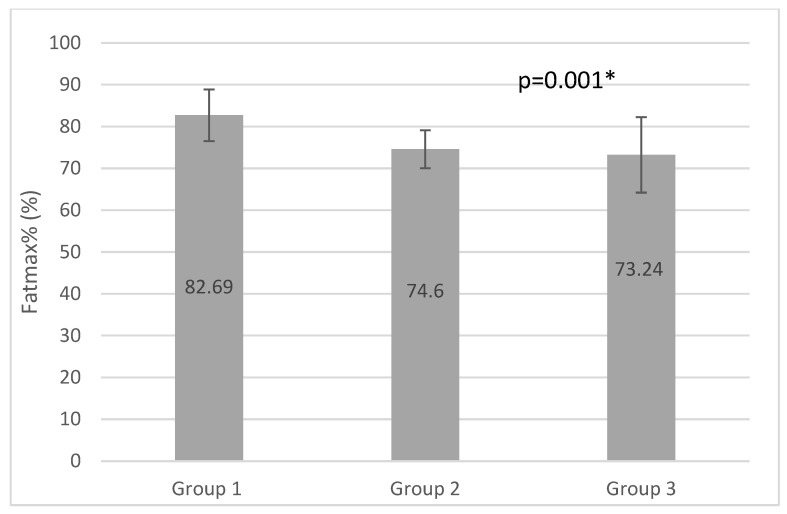
Fatmax% results of the groups (* *p* <0.05).

The results of our study show that participants in group 3, who fulfilled light aerobic training and resistance training only 2 times a week, had significantly lower VO2max, MET, and Fatmax% values than group1. (Figure 1, Figure 2 and Figure 3) The effects of aerobic training performed three times per week for 35–55 min in in group 2 lagged behind the development of group1, which trained with higher frequency and duration. Comparing the results of group2 and group3 we can see higher MFO, FatmaxHR, and VO2max results and significantly higher VO2max% in group 3, but significantly higher values in Fatmax% in group2.

According to the statistical analysis within the groups, there were no significant differences between the genders in terms of MET, VO2max, FatmaxHR, Fatmax%, and VO2max% (in group 1: *p*= 0.596, 0.984, 0.120, 0.722, and 0.678, respectively; in group 2: *p* = 0.503, 0.767, 0.203, 0.483, and 0.634, respectively; and in group 3: *p* = 0.126, 0.924, 0.540, 0.942, and 0.201, respectively) (*p* <0.05). Examining BMI values within the groups, we found significantly higher values of BMI among men in all groups (group1: *p* = 0.016, group2: *p* = 0.003, and group3: *p* = 0.002), but there was no significant difference between the three groups (0.119) (*p* < 0.05).

## 4. Discussion

### 4.1. Metabolism

Metabolic pathways during physical activity are determined by gender, nutritional status, diet, training status, and exercise intensity, duration, and modality [33,34]. The ability to use all of the possible energy pathways when needed is the key to a healthy metabolism. Carbohydrate oxidation is characteristic of higher exercise intensity; thus, fat oxidation is more important at lower intensities. During endurance training, energy is implemented by the oxidative metabolism of carbohydrate and fat [35]. During short intensive physical activity, the main energy resources are Creatine Phosphate (PCr) and glycogen [36,37,38]. During resistance training –according to involved muscle fiber type—intramyocellular triacylglycerol and glycogen content also decrease [39,40].

In our study, FatmaxHR is the heart rate at which maximal fat oxidation (MFO) occurs. The metabolic outcome of adapting to endurance training is the preference for fat oxidation by the oxidative phosphorylation pathway. 

Maximum Fat Oxidation (MFO) occurred at higher relative and absolute maximal heart rate values (FatmaxHR and Fatmax%) in group1. Group1 reached MFO at the highest VO2max% values; 66.7%, while group 3 reached 56.29% and group 2 48.13%, which was significantly lower than in Group1 (*p* = 0.00). Comparing the results of group 2 and group 3, we can see higher MFO, FatmaxHR, and VO2max results and significantly higher VO2max% in group3, but significantly higher values in Fatmax% in group 2. A correlation between respiratory capacity and maximal fat oxidation can be observed, as trained individuals have a better ability to oxidize fat at higher intensity of physical activity [41,42]. Stisen et al. examined endurance-trained and untrained women by a progressive ergometer cycling test and found a higher fat oxidation rate among endurance-trained women at moderate intensity (45–60% of VO_2_ max). MFO occurs at higher relative exercise intensity among those trained (56% vs. 53% of max intensity) [43]. Rosenkilde found high and moderate endurance training effective in increasing peak fat oxidation, as well as VO2max peak among sedentary overweight men [44]. Nodby et al. concluded in their paper that MFO (250+/−25 and 462+/−33 mg/min) was higher (*p* < 0.05) and occurred at a higher (*p* < 0.05) relative workload (43.5+/−1.8% and 49.9+/−1.2% VO (2max)) in trained than untrained subjects [45,46].

LeMura et al. compared resistance training, aerobic training, and mixed aerobic-resistance training with control groups during 16 weeks of training and 6 weeks of detraining. Results showed significant changes in values connected to fat metabolism, in the aerobic training group (ATG), similar to our findings. The resistance, mixed, and control groups did not show any significant changes in VO2max or body composition during the training and detraining periods, contradicting our findings. Other findings from the LeMura et al. study indicate that aerobic training can effectively increase the metabolic health and VO2 max of young women. It is essential to note that each of the alterations in the ATG disappeared after the 6-week detraining period [47]. We consider it essential to examine long-term metabolic and cardiorespiratory changes, thus we chose to evaluate the effects of 6 months of training.

Participants in our research were classified into groups based on the type and frequency and volume of their regular training. A total of 50% (group1), 35% (group2), and 76% (group3) of participants in each respective group were women. These results show mixed training (aerobic and light resistance training) to be the most preferred physical activity among the woman participants of the studied groups. 

### 4.2. VO2max

Our results showed significantly higher values of VO2max in group1, which can be explained by the chronic adaptation to the high frequency and long duration low intensity training (four times a week, 60–90 min) [48,49]. We were unable to undertake a comparative study of the groups’ values before and after six months of training since we only collected data once after the COVID-19 closures; thus, we compared the groups’ VO2 max and MET values to the standard values. Edvardsen et al. in their study established reference values for VO2max (absolute, relative to body weight and lean body mass) and other cardiorespiratory and metabolic parameters during maximum treadmill activity, in a large population ranging in age from 20 to 85 years. The authors suggested using findings as reference variables during treadmill cardiopulmonary testing. In group 1 and group 2 each participant had higher VO2max values than the physically inactive population normative, described by Edvardsen et al. [50] The statistical analysis revealed that men and women in group 1 had significantly higher VO2max values than the physically inactive population of the same age group (30–39 men and 30–39 women) (*p* = 0.001 and 0.034).

### 4.3. MET

Calculating the MET is a clear and practical method for expressing the energy expenditure of physical activities as a multiple of the metabolic rate at rest. Energy expenditure values for a variety of home and recreational activities are determined in METs, and the grade-by-grade intensity levels of different exercise treadmill protocols can be compared. Despite its limitations, using MET offers a simple way to describe an individual’s functional capability or exercise tolerance [51]. We found the highest MET values in group 1 (14.24), which was higher than in group 2 (13.12) and significantly higher than in group 3 (11.44, *p* = 0.046). However, these results suggest an average cardiorespiratory fitness level and a good health prognosis for all three groups [51]. 

## 5. Conclusions

Our research revealed that the studied training types and frequencies can be a proper tool for improving physical fitness. We conclude that aerobic training performed four times per week for 60–90 min increases fat metabolism better than aerobic training performed less frequently, or light resistance training and aerobic training mixed. Mixed training leads to greater MFO, FatmaxHR, and Fatmax% values than three sessions per week of aerobic exercise alone; therefore, we may assume that frequent resistance training is recommended. These findings suggest that regular physical exercise performed without the supervision of an expert has beneficial effects on the metabolism and cardiorespiratory system.

## 6. Limitations

Despite the significant impact of diet on metabolism and exercise metabolism, we were unable to analyze the nutritional habits of the subjects. We didn’t have the opportunity to evaluate body composition, which could have an effect on the results. The self-reported information, such as the frequency and length of the training could not be validated. Despite the lack of baseline data, population-based study data allowed us to compare the data of our experimental groups with the standard values of MET and VO2max, which can cause possible bias, but during the restrictions, it was impossible to measure them in person before the training; therefore, we conducted our experimental research with a single test following the lockdowns.

A follow-up design with repeated measurements on the same sample or with those who trained with professional supervision, or have not been subjected to any training would give more robust results. 

## Figures and Tables

**Table 1:** The demographic characteristics of the groups.

		Group 1 (*n* = 18)	Group 2 (*n* = 14)	Group 3 (*n* = 13)	Total (*n* = 45)	Sig
Physical activity	Type	aerobic training (cycling, running)	aerobic training (cycling, running)	aerobic, resistance training		
	Duration/training	60–90 min	35–55 min	45–60 min		
	Frequency/week	4	3	2		
Gender	Women	9	5	10	24	
	Men	9	9	3	21	
Age	Mean	37.6	40.28	34.78	36.34	0.710
	±SD	9.42	8.31	10.9	9.7	
Body mass (kg)	Mean	68.8	71.57	73.58	71.52	0.802
	±SD	12.97	9.4	21.63	14.40	
Height (cm)	Mean	174.6	176.0	171.83	174.42	0.351
	±SD	7.89	6.42	9.04	7.68	
BMI	Mean	22.86	22.79	25.00	23.48	0.119
(kg/m^2^)	±SD	2.63	1.74	4.85	3.31	

(*p* <0.05).

**Table 2:** The results of the incremental treadmill test.

		Group 1 (*n* = 13)	Group 2 (*n* = 14)	Group 3 (*n* = 18)	Total (*n* = 45)	Sig
MaxHr (Bpm)	Mean	183.46	184.33	191.33	185.91	0.233
	±SD	14.44	8.60	15.35	12.84	
RER (VCO2/VO2)	Mean	1.18	1.19	1.19	1.19	0.872
	±SD	0.07	0.09	0.10	0.09	
RER1 (Bpm)	Mean	169.00	162.27	168.50	166.17	0.423
	±SD	17.16	12.50	19.46	16.10	
VO2max (mL/kg/min)	Mean	50.19	45.97	41.39	46.15	0.045 *
	±SD	10.85	5.04	10.35	9.26	
Load (Watt)	Mean	405.87	337.78	303.83	351.42	0.082
	±SD	171.35	66.03	99.25	123.26	
MET	Mean	14.24	13.12	11.44	13.04	0.046 *
	±SD	3.42	1.47	3.44	2.95	
MFO (g/day)	Mean	881.87	817.50	920.08	866.31	0.715
	±SD	335.19	404.19	251.99	341.45	
FatmaxHR (Bpm)	Mean	153.13	137.44	142.17	143.93	0.004 *
	±SD	13.52	8.79	16.05	14.12	
Fatmax% (%)	Mean	82.69	74.60	73.24	76.94	0.001 *
	±SD	6.17	4.54	9.02	7.59	
VO2max% (%)	Mean	66.7	48.13	56.29	56.72	0.000 *
	±SD	10.92	11.93	12.67	13.5	

Maximum heart rate (HRmax), Respiratory Exchange Ratio, RER, Heart rate at RER 1 (RER1), maximum VO2 (VO2max), Load, metabolic equivalent (MET), Maximum fat oxidation (MFO), Heart rate at MFO (FatmaxHR), maximum heart rate percentage at MFO (Fatmax%) and maximum VO2max percentage at MFO (VO2max%) (* *p* < 0.05).

**Table 3:** Significant results of Bonferroni and Scheffe analysis.

Dependent Variable	(I)	(J)	Mean Diff (I–J)	Std Error	Sig	95% Confidence Intervall	
VO2max (mL/kg/min)							
Scheffe	Group 1	Group 2	4.22	3.08	0.399	−3.59	12.03
		Group3	8.80 *	3.41	0.046 *	0.14	17.46
Bonferroni							
	Group 1	Group 2	4.22	3.08	0.534	−3.46	11.90
		Group 3	8.80 *	3.41	0.041 *	0.29	17.31
MET							
Scheffe	Group 1	Group 2	1.12	0.98	0.525	−1.37	3.61
		Group 3	2.80 *	1.08	0.046 *	0.03	5.56
Bonferroni	Group 1	Group 2	1.12	0.98	0.778	−1.32	3.57
		Group 3	2.80 *	1.08	0.041 *	0.08	5.51
FatmaxHR (Bpm)							
Scheffe	Group 2	Group 1	−15.68 *	4.41	0.004 *	−26.90	−4.48
Bonferroni	Group 2	Group 1	−15.68 *	4.41	0.003 *	−26.71	−4.67
Fatmax% (%)							
Scheffe	Group 2	Group 1	8.08 *	2.27	0.004 *	−13.86	−2.31
		Group 3	9.44 *	2,52	0.002 *	−15,84	−3.04
Bonferroni	Group 2	Group 1	8.08 *	2.27	0.003 *	−13.76	−2.41
		Group 3	9.44 *	2.52	0.002 *	−15.73	−3.16
VO2max(%) (%)							
Scheffe	Group 1	Group 2	18.57	2.43	0.000 *	−3.54	12.54
Bonferroni	Group 1	Group 2	18.57	2.43	0.01 *	−3.54	12.48

(* *p* < 0.05).

## Data Availability

The dataset supporting the conclusions of this article is available from the corresponding author on reasonable request in accordance with MDPI Research Data Policies.

## Abbreviations

FatmaxHR	Heart rate at Fatmax
Fatmax%	Percentage of maximum heart rate at Fatmax
HRmax (Bpm)	Maximum heart rate
IC	Indirect Calorimetry
IPAQ-HL	International Physical Activity Questionnaire
IR	Insuline resistance
Load (Watt)	Maximum load
MFO (g/day)	Maximal fat oxidation during exercise
MET	Metabolic Equivalent of Task
MF	Metabolic flexibility
MFO	Maximal fat oxidation
MS	Metabolic Syndrome
PA	Physical activity
PCr	Creatine phosphate
RER	Respiratory Exchange Ratio
RER1 (Bpm)	Heart rate at 1RER
VO2max (mL/kg/min)	Aerobic capacity
VO2max%	The percentage of VO2max at MFO
